# A Scoping Review of Assessments in Undergraduate Medical Education: Implications for Residency Programs and Medical Schools

**DOI:** 10.1007/s40596-025-02136-4

**Published:** 2025-04-01

**Authors:** Xiaomei Song, Elle Cleaves, Ellen Gluzman, Biana Kotlyar, Rachel A. Russo, David C. Schilling, Carol Ping Tsao, James C. West

**Affiliations:** 1 Case Western Reserve University School of Medicine, Cleveland, OH USA; 2 Uniformed Services University, Bethesda, MD USA; 3 Temple University Lewis Katz School of Medicine, Philadelphia, PA USA; 4 Rosalind Franklin University of Medicine and Science Chicago Medical School, Chicago, IL USA; 5 VA San Diego Healthcare System, San Diego, CA USA; 6 Loyola University Chicago Stritch School of Medicine, Chicago, IL USA; 7 Medical College of Wisconsin, Milwaukee, WI USA

**Keywords:** Undergraduate medical education, Assessment, Competency-based education, Validity, Reliability

## Abstract

**Objective:**

Assessment at medical schools plays a crucial role by providing feedback, monitoring student promotion, and informing resident selection. Limited research has been conducted to synthesize key features of assessments, and even less is known about how these studies sought validity and reliability evidence. A scoping review was performed to explore key features of assessments and their validity and reliability evidence.

**Methods:**

Various databases were searched ranging from 2004 to April 2021 following PRISMA guidelines. In-depth reviews of the full text were performed on all selected empirical studies during the extraction phase.

**Results:**

The final analysis included 218 empirical studies. Various tools were identified, ranging from traditional multiple-choice questions to more contemporary tools incorporating technology and more contextualized workplace-based assessment. Patient care and medical knowledge were the most frequently assessed, primarily using the quantitative methodology. These studies often adopted traditional perspectives in collecting validity evidence based on relations to other variables and internal structure. Many of them used a narrow conceptualization of validity, with some failing to reference validity at all.

**Conclusions:**

There was no single assessment that could easily differentiate medical students in a standardized, meaningful way. Despite the existence of contemporary validity theories for over two decades, there remains a need for greater education regarding the pivotal role of validity in conducting assessment research. While psychiatry and other residency programs will continue to face challenges in differentiating applicants, these changes present opportunities for medical educators and schools to validate assessments that are highly contextualized to their specific educational environments.

Assessment at medical schools plays a crucial role by providing feedback, monitoring student progress, ensuring that students achieve intended competencies, and supplying information for resident selection. The literature in medical education assessment is characterized by a diverse array of assessments, which are scattered across various fields and multiple medical specialties. These tools assess varying competencies and serve various purposes that aligned with specific goals, requirements, and available resources [1–3]. These key features are often guided by test specifications (also called test blueprints), which include documentation of the purpose and intended uses of the assessment, as well as detailed decisions about content, format, length, scoring, and score reporting. In addition, the robustness and quality of these assessments has been examined widely. Central to the quality of assessments is the concept of validity, which has evolved over time. Contemporary theory views validity as a unitary concept requiring ongoing, situated judgments to support the intended interpretation of test scores for the proposed use [4–6]. The *Standards for Educational and Psychological Testing* endorsed by over 50 professional associations and organizations (e.g., Prometric, NBME, ABIM) outline five sources of evidence that might be used in evaluating the validity of a proposed interpretation of assessment scores for a particular use [7]. These sources include evidence based on content, response processes, internal structure, relations to other variables, and consequences (see Table 1).

**Table 1:** Extraction list: definitions and frequencies

Types of assessments	Definitions	No. of studies (%)
Non-WBAs	Assessments conducted in classroom setting, not in authentic workplace or simulated setting	121 (56%)
• Institute-specific MCQs	Written examination developed and administered at a school/program level using recall/vignette-based questions with answer choices based on a dichotomous (correct/incorrect) or weighted partial scoring system (e.g., Script concordance test)	45 (16%)
• National MCQs	Standardized written examination developed and administered at a national level often used across different institutes	74 (34%)
• Performance Assessments	Performance-based written or oral examination with open-ended questions which is assessed based on assessment criteria across a varying degree of achieved levels (e.g., oral exam, interview, short answer questions)	29 (18%)
• Attribute and perception surveys	Self-reported information to gather insights into an individual's attributes, perceptions, and attitudes (e.g., empathy, emotional intelligence, situational awareness, and personality traits)	9 (4%)
Semi-WBAs	Assessments conducted in simulated, controlled setting	88 (40%)
• OSCE/Clinical skills assessments	Performance-based assessment using a series of stations where students rotate through interactions with SPs about simulated clinical tasks in a controlled and simulated clinical environment	72
• Technology-enhanced simulation assessments	Performance-based assessment, wherein students physically interact with a tool or device that mimics clinical care (e.g., high-fidelity, virtual reality patient)	18
WBAs	Assessments conducted within authentic clinical environments where patient care is delivered	110 (51%)
• Institute-specific clinical performance assessments	Performance-based assessment developed and administered at a school/program level based on assessment criteria across a range of achieved levels in the workplace environment	86 (39%)
• Mini-CEX	Performance-based assessment using the Mini-Clinical Evaluation Exercise	11
• RIME	Performance-based assessment in the workplace environment using the Reporter, Interpreter, Manager, and Educator (RIME) model	6
• EPAs-based assessments	Performance-based assessment of Entrustable Professional Activities in the workplace environment	6
MSPE/Dean’s Letter/Standardized Letter of Evaluation	A standardized letter from the institute or faculty members about a student’s salient experiences, attributes, and academic performance	8 (4%)
Letter of recommendations	A non-standardized letter from faculty members or other individuals about a student’s salient experiences, attributes, and academic performance	6 (3%)
Other	Does not fall in the above assessment categories (e.g., honors, AOA, class rank, faculty perceptions of assessments)	26
Focused competencies	Definitions	No. of studies (%)
Patient care	Assessing the area of patient-centered care for the treatment of health problems and the promotion of health	118 (54%)
Medical knowledge	Assessing knowledge of biomedical, clinical, epidemiological and social-behavioral sciences, as well as the application of this knowledge to patient care	107 (49%)
Practice-based learning	Assessing the ability to investigate and evaluate one’s care of patients, to appraise and assimilate scientific evidence, and to continuously improve patient care based on constant self-evaluation and life-long learning	18 (8%)
Interpersonal and communication skills	Assessing interpersonal and communication skills that result in the effective exchange of information and collaboration with patients, their families, and health professionals	56 (26%)
Professionalism	Assessing commitment to carrying out professional responsibilities and an adherence to ethical principles	37 (17%)
System-based practices	Assessing awareness of and responsiveness to the larger context and system of health care, as well as the ability to call effectively on other resources in the system to provide optimal health care	12 (6%)
Comprehensive	Assessing multiple individual competencies (e.g., generically used and not clearly defined)	74 (34%)
Other	Does not fall in the above competencies (e.g., empathy, emotional intelligence, situational awareness, and personality traits)	12 (6%)
Research methodology	Definitions	No. of studies (%)
Quantitative	The collection and analysis of numerical data to measure variables and conduct simple and complex statistical analyses including descriptive and inferential statistics using techniques such as Generalizability theory, regression, and factor analysis	191 (88%)
Qualitative	The collection and analysis of non-numerical, narrative data to conduct in-depth exploration, interpretation, and understanding of beliefs, attitudes, behaviors, or social interactions using techniques such as interviews, focus groups, or document analysis	3 (1%)
Mixed-method	The collection and analysis of both numerical and non-numerical data within a single study to provide a complementary or comprehensive understanding of the research topic	24 (11%)
Sources of validity and reliability evidence	Definitions	No. of studies (%)
Content	Evidence of the adequacy between content of assessment method and construct of interest	33 (15%)
Response process	Evidence of the fit between the construct and the detailed nature of the performance or individual response engaged in by students or assessors	23 (11%)
Internal structure	The degree to which individual items within the instrument fit the underlying constructs	103 (47%)
• Reliability	Consistency over items (e.g., Cronbach’s alpha)	76 (35%)
	Consistency over judges (e.g., inter-rater reliability [kappa])	61 (28%)
	Consistency over time (e.g., intra-rater)	4 (2%)
Relationship to other variables	Analyses of the relationship between assessment scores and other variables such as correlation, prediction, and criterion	136 (62%)
Consequences	Consequences and impacts of proposed interpretations of assessments for their intended uses	12 (5%)

Although much research has been conducted to describe and examine individual assessments used in undergraduate medical education, few studies have synthesized the key features of these tools and even less is known about how these studies sought validity and reliability evidence [2]. In addition, the elimination of USMLE Step 2 CS and the shift of Step 1 to a Pass/Fail system have had a profound impact on the landscape of the US medical education assessment [8, 9]. Consequently, medical associations have intensified their efforts to identify effective methods for evaluating the competence of medical students when it comes to selecting candidates for residency programs. The Coalition for Physician Accountability convened the Undergraduate Medical Education to Graduate Medical Education Review Committee (UGRC), recommending the development of meaningful and robust assessments for evaluating medical students’ knowledge and skills as they transition to residency [10]. The long-standing search for a more holistic and competency-based assessment approach for residency program candidate selection has been fast tracked due to these changes. Recognizing these challenges, the Association of Directors of Medical Student Education in Psychiatry (ADMSEP) in the USA formed a task force in July 2020 to determine best practices in assisting program directors to differentiate applicants without a Step 1 score. As such, this study was conducted to identify quality performance metrics that offer program directors a means of distinguishing the achievements of applicants. The project started as an offshoot of a task force endorsed by the organization and proceeded as a subcommittee under the task force. This manuscript represents the collective findings and recommendations of the task force. Its methodological approach and preliminary results have been presented at the organization’s annual conferences.

To achieve these goals, we conducted a scoping review of current literature, examining empirical, peer-reviewed studies on available methods for assessing medical student performance. We chose a scoping methodology because preliminary searches had revealed a complex and heterogeneous body of literature, and our research question was exploratory [11, 12]. We report on the empirically researched assessments, but we do not seek to be exhaustive. Using the scoping review, this study examined the broad field of assessment features with particular focus on validity and reliability evidence. By examining features of assessments used in medical education systematically and investigating validity evidence supporting these tools, we aim to provide educators, researchers, and policymakers with a solid foundation for further advancements in this field. Specifically, the study intended to answer the following questions:

1. What are the major types of assessments in medical education?
2. What competencies do assessments intend to assess?
3. What specific sources of validity and reliability evidence are typically reported by using what methodology?

## Methods

**Fig. 1:**
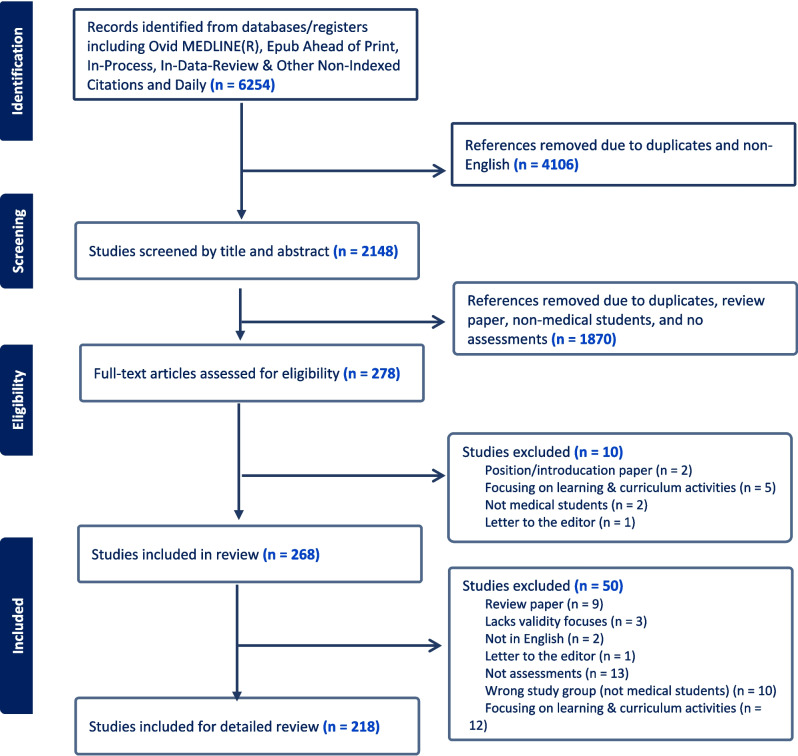
PRISMA (Preferred Reporting Items for Systematic Reviews and Meta-Analyses) diagram

The review was conducted following the PRISMA standards of quality for reporting systematic reviews (see Fig. 1). An experienced research librarian designed the search strategy and various databases were searched ranging from 2004 to April 2021 to capture the most up-to-date publications available at the time to the review. The librarian ran the search in Ovid MEDLINE(R) and Epub Ahead of Print, In-Process, In-Data-Review, and Other Non-Indexed Citations and Daily. A combination of Medical Subject Headings (MeSH) and keywords was used. The search used various terms regarding medical students and assessment. Terms used for medical students include clinical clerkship, undergraduate medical education, medical students, medical trainee, clinical student, clinical education, clinical elective, and clinical rotation. Terms for assessment include educational measurement, professional competence, clinical competence, assessment, grading, OSCE, clinical skills exam, and objective structured clinical examination. We limited the search parameters to English language, articles published since 2004 when Step 2 CS came into existence and changed much of teaching and assessments. Articles pertaining to non-medical programs like nursing, dental, and pharmacy education were excluded, as were position papers, review papers, and empirical papers focusing on curriculum changes or innovative instructional/learning approaches.

The ADMSEP task force members, including eight members at any given time, reviewed the empirical articles throughout various stages using Covidence. First, the group divided into pairs to review articles using the inclusion and exclusion criteria (see Fig. 1). Each member of the pair reviewed the titles and abstracts independently, and then the pair met to compare and reach consensus about including or excluding the article for further consideration. After that, full-text articles were assessed by two independent reviewers for key elements of the individual article. Interrater reliability was assessed using exact agreement at this level. Following this, the task force members discussed the definitions and extraction template extensively. We piloted a template with eight papers as a whole team in order to establish clear definitions for each aspect of the form (see Table 1). We refined the data abstraction form through iterative reviewing and numerous revisions until reaching saturation. During the extraction phase, two members reviewed the empirical studies independently using the final data abstraction form. The discrepancies that arose between two independent reviewers were reconciled in order to achieve consensus for each individual paper. The two reviewers reconciled their disagreement between themselves. Considering the research questions of this scoping review, the quality of validity and reliability evidence was not formally assessed.

The data abstraction form and in-depth reviews of the full text on all selected empirical studies were informed by the literature. Specifically, the types of assessments were informed by the classifications identified by Daniel and her collaborators: non-workplace-based assessment (non-WBAs), assessment in simulated clinical environments (semi-WBAs), and workplace-based assessment (WBAs) [5]. Regarding focused competencies, we adopted the six domains of competence as the foundational reference list: Patient Care (PC), Medical Knowledge (MK), Professionalism, Interpersonal and Communication Skills (ICS), Practice-based Learning and Improvement (PBLI), and Systems-based Practice (SBP). These six domains are widely used and researched [13]. For the purpose of this study and following deliberate discussion, Step 1 was coded as MK, PC, ICS, and PBLI as NBME specifies. Step 2 was coded as MK, PC, professionalism, and SBP. NBME Subject Exam was coded as MK and PC. Research methodologies were classified as quantitative, qualitative, and mixed methods. Finally, we used five sources of validity evidence, as summarized in the *Standards of Educational and Psychological Testing*, to inform data synthesis [7]. While some studies focused on one single essential tool, most studies used multiple assessments. We examined both primary and secondary assessments, particularly when these studies explored relationships with other variables such as correlation and prediction.

## Results

As Fig. 1 shows, the search parameters and inclusion/exclusion criteria yielded a total of 6254 articles. Title and abstract review excluded 4106 articles based on the inclusion/exclusion criteria. The remaining 2148 articles were then tagged for eligibility in the full-text review. A total of 268 full-text empirical studies were assessed, and ultimately, 218 articles were included for detailed review. Regarding the inter-rater agreement, judgments about the overall favorability of a full-text review to be included in the final review were in exact agreement 73% of the time.

The included articles came from many different countries; however, the majority came from the USA, Europe, and Canada. Up until 2014, the examination of validity evidence was limited every year, with only a single-digit number of studies (excluding the year 2011, which had 14 papers). Nevertheless, there has been an exponential growth in the number of empirical papers related to validity, reaching 38 in 2017 and 31 in 2018. In the following, we discussed four major areas: (1) types of assessments, (2) focused competencies, (3) research methodology, (4) sources of validity and reliability evidence under investigation. Table 1 presents the extraction list including definitions and frequency of the studies in the relevant area.

### Types of Assessments

Five major types of the assessments emerged including non-WBAs, semi-WBAs, WBAs, MSPE/Dean’s Letter/Standardized Letter of Evaluation (SLOE), and Letter of recommendation (LOR). The first type of non-WBAs includes four subcategories: Institute-specific multiple-choice questions (MCQs), National MCQs, Performance assessments, and Attribute and perception Survey. The second type of semi-WBAs includes OCSE/Clinical Skills Examinations and Technology-enhanced simulation. The third type of WBAs includes four subcategories: Institute-specific clinical performance assessments, Mini-Clinical Evaluation Exercise (Mini-CEX), Reporter, Interpreter, Manager, Educator (RIME), and Entrustable Professional Activities-based (EPAs-based) assessments.

Overall, Non-WBAs remain the major type (*n* = 121, 56%) followed by WBAs (*n* = 110, 50%) and semi-WBA tools (*n* = 88, 39%). Only eight empirical studies examined MSPE/SLOE and six examined LOR [14]. Twenty-six papers were coded as Other such as honor distribution, premedical GPA, MCAT score, class rank, Alpha Omega Alpha membership, advanced degrees, awards, volunteer activities, residency match rate, research experiences, first author publications, career choice, and concerns (“red flags”) in performance evaluations [15].

Within the non-WBA category, national standardized MCQ exams such as Subject and Step exams were widely used (*n* = 74, 34%) [16]. Assumed to be valid tools, they were often referenced to examine correlations with overall grades or as predictors of success investigated by many schools. Medical programs also developed various school-specific MCQs (*n* = 45, 16%) to examine student performance [17]. Among these, there were 11 Script Concordance Test (SCT) to assess clinical reasoning and probe the multiple judgments that are made in the process with standardized grading criteria. These studies were mostly conducted in France, Australia, and Canada [18]. In addition, 29 studies (18%) examined performance assessments by using oral exam, interview, short answer/essay-based questions, or observations, mostly focusing on clinical reasoning [19]. Nine studies (4%) examined individual’s attributes, perceptions, and attitudes such as emotional intelligence or personality using survey questionnaires with self-reported or peer/faculty-reported data [20]. These studies often investigated how positive and/or negative personality traits impacted assessment and achievement.

Medical programs used a variety of different tools to assess clinical performance. While some program adopted or modified a national tool such as Mini-CEX (*n* = 11, 5%), RIME (*n* = 6, 3%), or EPA-based assessment (*n* = 6, 3%), a majority of medical schools made efforts to examine school-specific clinical tools (*n* = 86, 39%) [21–23]. While some school-specific tools examined achievements more broadly, some focused on specific areas such as history and physical exams, manual dexterity, or procedural skills in the workplace environment (often in surgery) [24].

Finally, 72 studies (33%) used OSCE or Clinical skills examinations and 18 studies (8%) adopted technology-enhanced simulation assessments such as high-fidelity and virtual reality patients (*n* = 18) in a simulated, controlled-clinical environment [25, 26]. Although specific stations and grading criteria differed between schools, they focused on clinical skills and communications.

### Focused Competencies

In addition to using the ACGME’s six domains of competence for the analysis, the scoping review also included the Comprehensive and Other categories. Within the comprehensive category, a substantial portion of the studies (*n* = 74, 34%) examined clinical skills broadly, lacking explicit focus on specific competencies (e.g., RIME, clinical performance, rounding, clinical reasoning) [27]. Additionally, 12 papers (6%) were classified as Other. These papers did not directly examine competencies; instead, they focused on areas such as grade distributions, the cognitive processes of raters, or letters of recommendation [28]. Overall, not all studies explicitly articulated their focus on competency, nor were they structured within the framework of competency-based education. Some explored concepts like personal characteristics, empathy, and emotional intelligence [29].

Due to the highly interwoven nature of these six competencies, the studies often assessed multiple competencies and domains. Not surprisingly, PC (*n* = 118, 54%) and MK (*n* = 107, 49%) were the two competencies which were mostly frequently assessed [30]. Of the 118 studies which investigated patient care, only 14 papers concentrated on PC as the sole competency being assessed, primarily focusing on surgical skills [31]. Among 56 papers (26%) which examined ICS, the studies focused on assessment of students’ written notes and clinical presentations, and only 9 papers had just ICS as the sole competency being assessed [32]. A total of 37 papers (17%) examined Professionalism in terms of a commitment to carrying out professional responsibilities and an adherence to ethical principles. Among them, 8 papers had professionalism as the main topic of the paper, and the other papers discussed professionalism as part of the grading scheme or assessment [33]. Finally, PBLI (*n* = 18, 8%) and SBP (*n* = 12, 6%) were comparatively under-researched. Even when the studies examined these two competencies, they often assessed multiple competencies simultaneously or used them as secondary tools, thereby providing limited insights into their specifics [34, 35].

### Research Methodology

The vast majority of studies (*n* = 191, 88%) employed simple and/or complex quantitative methods, encompassing a wide range of techniques. These methods ranged from basic descriptive analyses, providing foundational insights into the data, to advanced statistical methods such as correlation and regression analysis, factor analysis, structural equation modeling, and Generalizability theory [36, 37]. A very smaller portion of studies (*n* = 3, 1%), all conducted in the recent 10 years, adopted qualitative approaches only, including focus groups, retrospective think-aloud, and one-on-one interviews [38]. Additionally, a few studies (*n* = 24, 11%) adopted the mixed-methods approach, integrating both quantitative and qualitative methodologies, which encompassed techniques like Delphi studies [39, 40]. Using various designs (e.g., explanatory, concurrent), the mixed-methods approach aimed to achieve a more comprehensive understanding of the research topic by leveraging the strengths of both quantitative and qualitative data.

### Sources of Validity and Reliability Evidence Under Investigation

As mentioned previously, our analysis regarding the sources of validity evidence was guided by the *Standards*, including evidence based on test content, response processes, internal structure, relations to other variables, and consequences of testing [7]. While some studies used a more limited validity framework such as construct or predictive validity, some studies, earlier ones in particular, did not mention anything related to validity [41, 42]. The two most frequently investigated sources of validity evidence were relations to other variables (*n* = 136, 63%) and internal structure (*n* = 103, 47%). The validity evidence based on relations to other variables has been extensively examined, yielding much rich, in-depth information. Over time, these investigations delved into diverse areas including predictive, concurrent, convergent, and discriminant evidence among different populations (e.g., novice medical students vs. experienced attendings) [43, 44]. The studies examined a wide range of assessment results, ranging from pre-matriculated data (MCAT scores, pre-medical school GPA, MMI interviews, etc.) to pre-clerkship performance (various course grades, Step 1 results, OCSEs, etc.), clerkship (Subject scores, honors, rank order, etc.), and onward to residency match and resident performance (residency interviews, program director assessments, specialty certifying exams, etc.). Residency programs and medical schools made significant efforts to identify successful residents or provide resources in supporting at-risk students.

Similarly, the validity evidence of internal structure has been widely researched for a long time. These studies examined the distribution of student performance, analyzed inter-item correlation or discrimination, explored the internal structures of assessments, and investigated reproducibility across different items, stations, raters, or versions of the test [45, 46]. While there is a general consensus among researchers that reliability is considered a necessary but not sufficient condition for validity, only about half of the studies reported or made inferences about reliability estimates. Specifically, 76 studies reported consistency over items (e.g., Cronbach’s alpha), 61 examined consistency over judges (e.g., inter-rater reliability [kappa]), and 4 studies reported consistency over time (e.g., intra-rater) [47, 48]. Notably, due to wide endorsement and the unavailability of NBME item-level information, the studies utilizing NBME often did not provide reliability information.

There was a significant decrease in the number of studies reporting other sources of validity evidence. Only 33 studies examined content validity evidence, 23 explored response processes, and 12 investigated the consequences of testing. Regarding content validity evidence, the studies primarily centered on group consensus using Delphi techniques, development of test blueprints, or iterative instrument development processes [49, 50]. In terms of response processes, the studies typically involved analyzing the verbal descriptions of thought processes provided by both raters and test takers, as well as examining instances of disagreement among raters regarding scoring [51, 52]. Consequential validity was the least explored area. The studies often employed standard setting methodologies to determine passing or honors thresholds and investigated the impact of assessments on students’ learning and career choices [53–55].

## Discussion

A diverse array of assessments was identified, ranging from traditional MCQs to more contemporary methods incorporating technology and more contextualized workplace, school-based assessment. Non-WBAs, especially national standardized exams, continue to be prevalent, underscoring their popularity for their objectivity and usefulness for national or institution-wide comparisons. In addition, the use of WBAs signify a value towards assessing learners based on demonstrated performance in authentic clinical settings. The adoption or modification of standardized WBAs such as Mini-CEX, RIME, or EPA-based assessments exemplifies the richness and diversity of contemporary assessment approaches. These frameworks offer mechanisms for assessing student competencies while accommodating the varied assessment frameworks, contexts, and requirements of different medical programs. Finally, the prominence of semi-structured assessments suggests a growing emphasis on a controlled environment to assess clinical competence and communication. These simulated assessments provide a safe space for learners to practice decision-making and technical skills, bridging the gap between medical knowledge and practical application [1]. Overall, these findings underscore the importance of maintaining a balanced approach in medical education assessment. Medical schools should incorporate various types of assessments, including non-WBAs, semi-WBAs, and WBAs, using both standardized and institute-specific assessments. By diversifying assessment methods, medical school can reduce the risk of bias inherent in any single tool, such as cultural, gender, or methodological biases that may influence grading and performance. Incorporating a variety of tools and formats ensures a more comprehensive assessment of student competencies, fosters inclusivity, and supports the validity and reliability of assessment outcomes.

Competency-based medical education (CBME) has been increasingly used since the 2000s. Many medical schools and residency programs around the world have transitioned to CBME to ensure that physicians are equipped with the essential competencies to meet the evolving demands of healthcare delivery. However, the scoping review found that some studies neither clarified which competencies the assessment intended to examine, nor were they conducted within the ACGME 6-domain framework (e.g., personality), reflecting gaps in using or defining competencies [56]. Among these six domains, it is not surprising that patient care and medical knowledge are the most frequently assessed domains. This emphasis reflects the foundations of medical practice, highlighting the importance placed on clinical competence and theoretical understanding in medical education. In contrast, system-based practices and practice-based learning were least assessed. The underassessment of these domains may be attributed to various factors, such as the constructs overlapping with other competencies, limited resources, time constraints, and a historical focus on medical knowledge and patient care in medical education. The current initiative in establishing a common set of foundational competencies and sub-competencies for undergraduate medical education may provide medical schools with clearer guidance on the CBME framework and encourage them to devote more efforts to address all competencies.

A large majority of studies under review used the quantitative methodology. While descriptive analysis provides foundational understanding of the data, more complex analysis such as prediction modeling and Generalizability theory enables researchers to build assessment rigor and infer relationships. Moreover, a notable portion of studies employed a mixed methods approach, which allows researchers to capitalize on the strengths of both quantitative and qualitative data, providing a more comprehensive understanding of the research topic. By triangulating data from multiple sources, researchers can corroborate findings, enhance validity, and offer deeper insights into complex phenomena. In contrast, very few studies used qualitative approaches solely. By delving into participants’ perspectives and narratives, qualitative research helps the understanding of the human elements involved in medical education and assessment and gains insights into complex social processes that quantitative data alone may not capture [57, 58]. The lack of qualitative approaches highlights the importance of recognizing the multifaceted nature of medical education and the need for diverse research methodologies to capture its complexity [59].

As described earlier, this scoping review found that some studies used a narrow conceptualization of validity and even neglected to reference any validity framework. The most common sources of validity evidence under investigation were these traditional approaches: relations to other variables and internal structure. Despite the existence of contemporary validity frameworks for over two decades, there remains a need for greater education among researchers and educators regarding the pivotal role of validity in medical education assessment [4, 5]. This study shows research efforts in exploring the relationship between assessment scores and other relevant variables, for example, correlations with the USMLE scores or predictive validity for residency selections. Similarly, the results on internal structure and reliability demonstrate attention to the analysis of individual responses, instrument consistency, and scoring procedures. Although reliability evidence serves as a crucial foundation upon which validity evidence can be built, the findings of this scoping review indicate a gap in the lack of the reporting or inferences of reliability evidence.

The other three sources of evidence—content, response process, and consequences—received less attention. Medical associations and schools have begun to focus on more content evidence, indicating a consideration of the alignment between the content of the assessment method and the construct of interest. It is also crucial to analyze rubric interpretations and grading responses, including actions, strategies, and thought processes of medical students and faculty raters. Additionally, the scarcity of empirical studies focusing on consequences suggests a potential gap in evaluating the broader impacts of assessments on teaching, learning, and educational practices [60]. Assessments should not only measure student competency but also inform instructional strategies, curriculum development, and learner support mechanisms. Therefore, there is an urgent need for greater attention to the consequences of assessments, including their influence on educational practices and the overall learning environment.

One limitation of this study is that the most recent literature was not included in the analysis. This exclusion was due to the time required to thoroughly analyze all the references uncovered in our study, which may have led to a potential lack of representation of the most current research findings in the field of assessment in medical education. There have been 1411 articles added to the literature since April 2021, which were not included in our analysis. The exclusion of these recent articles may have implications for the comprehensiveness and currency of the findings presented in this study, as newer research developments and insights may not be captured. Another limitation is that sources of validity evidence were not always presented with clarity and completeness in every paper. Although widely discussed, individual researchers had different ideas about the coding of national exams. Even so, there was substantial discussion among researchers and every effort was made to accurately categorize and analyze the identified articles. Finally, it is important to note that this study did not intend to appraise the quality of the empirical studies under investigation regarding validity and reliability evidence. Conducting such a thorough evaluation was beyond the scope of this study and would have required considerable time, expertise, and resources. Future studies may investigate research rigor and provide valuable insights into the quality of the findings derived from these studies.

In conclusion, the study provides important implications for medical assessment research, as well as for undergraduate medical programs and residency programs internationally. Regarding medical assessment research, this scoping review concluded that empirical studies examined a wide range of assessments focusing on various competencies, with certain competencies receiving more attention than others. These studies primarily concentrated on traditional perspectives in collecting validity evidence based on internal structure and relations to other variables, predominantly using the quantitative methodology. The findings emphasize the importance of educating medical education researchers and educators about contemporary validity theories.

In addition, results indicate that there is no one-size-fits-all, checkbox approach for resident selections because medical schools often use various types of assessments, both standardized and institute-specific, to address unique curriculums, expectations, objectives, and priorities. Although generalizability—where assessment results can be applied to different populations, settings, and contexts—is highly valued in medical education research, medical schools often adopt instructional and assessment methods tailored to the diverse needs of their student populations and consider variations in learning and teaching environments. Therefore, there was no single assessment that could easily differentiate medical students in a standardized, meaningful way, suggesting that residency program directors should continue to emphasize a holistic review of applicants.

While changes to USMLE will likely accentuate challenges in differentiating applicants, these changes present opportunities for medical educators and schools to develop contextualized assessments focusing on the accumulation of validity evidence. Medical schools must be transparent and take the opportunity to modify and validate assessments that are highly contextualized to their specific educational environments. Medical educators and researchers should prioritize efforts to accumulate validity and reliability evidence across all competency areas that students are required to achieve. It is vital to foster interdisciplinary collaboration and methodological diversity within the research community to further enhance the quality and rigor of assessment research. The recent community-building efforts by NBME serve as a good example. The NBME Speakers Bureau offers a complimentary knowledge network, connecting medical educators and schools with assessment experts. By investigating research questions from multiple sources, enriching research findings across all competencies, and ultimately contributing to the continuous improvement of medical curriculum and assessment, we can anticipate positive consequences for students, residency programs, and the healthcare system at large.

## Data Availability

The datasets generated during and/or analyzed during the current study are available from the corresponding author on reasonable request.

## Declarations

### Disclaimer

The opinions and assertions expressed herein are those of the author(s) and do not reflect the official policy or position of the Uniformed Services University of the Health Sciences or the Department of Defense.
